# Age-related nomograms for antral follicle count and anti-Mullerian hormone for subfertile Chinese women in Singapore

**DOI:** 10.1371/journal.pone.0189830

**Published:** 2017-12-14

**Authors:** See Ling Loy, Yin Bun Cheung, Marielle Valerie Fortier, Chiou Li Ong, Heng Hao Tan, Sadhana Nadarajah, Jerry Kok Yen Chan, Veronique Viardot-Foucault

**Affiliations:** 1 Department of Reproductive Medicine, KK Women’s and Children’s Hospital, Singapore, Singapore; 2 Duke-NUS Medical School, Singapore, Singapore; 3 Center for Quantitative Medicine, Duke-NUS Medical School, Singapore, Singapore; 4 Tampere Center for Child Health Research, University of Tampere and Tampere University Hospital, Tampere, Finland; 5 Department of Diagnostic & Interventional Imaging, KK Women’s and Children’s Hospital, Singapore, Singapore; Universite Clermont Auvergne, FRANCE

## Abstract

**Background:**

Antral follicle count (AFC) and anti-Mullerian hormone (AMH) are known as the most reliable markers of a woman’s ovarian reserve and are related to age. There is currently no specific local age-related centile charts for AFC and AMH. Therefore, we aim to examine the relationship between AFC and AMH with age and construct age-related nomograms among a subfertile Asian population.

**Methods:**

This is a study involving Chinese women who had their AFC and AMH measured as part of their subfertility screening from December 2010 until November 2014 in KK Women’s and Children’s Hospital, Singapore. Ordinary least squares regression analysis was used to estimate the relationship of AFC and AMH with age, while age-related AFC and AMH nomograms for the 3^rd^, 10^th^, 25^th^, 50^th^, 75^th^, 90^th^ and 97^th^ percentiles were produced using the lambda-mu-sigma method.

**Results:**

A total of 1,009 women, aged 26 to 44 year-old, were included. On average, the AFC and AMH decreased respectively by 0.79 follicle (95% confidence interval -0.93, -0.64) and 0.38 ng/mL (95% confidence interval -0.43, -0.32) per year of age. The age-related nomograms of AFC showed an approximately linear pattern, inversely correlated with age, regardless of the percentile. For AMH, the pattern is linear for the 75^th^ percentile and below but shows a slightly accelerating decline for the 90^th^ and 97^th^ percentile. Overall, there were large inter-individual variations in AFC and AMH up to about 40 year-old.

**Conclusion:**

The declines of AFC and AMH over age are mostly linear among subfertile Chinese women in Singapore. The age-related AFC and AMH nomograms could be used as a reference chart by fertility practitioners. However, future validation with longitudinal data is required.

## Introduction

Over recent years, measurements of ovarian reserve to predict future reproductive life have become critical for women because of increased female subfertility due to postponement of childbearing [1]. There are varieties of ovarian reserve tests that include ultrasound and biochemical parameters [2]. Antral follicle count (AFC) and anti-Mullerian hormone (AMH) have been shown to be the best markers of ovarian reserve [2]. The AFC, measured by transvaginal ultrasound, describes the total number of follicles at ≥2mm in diameter that are observed during an early follicular phase [2, 3]. The AMH, a dimeric glycoprotein member of the transforming growth factor beta-family, is produced by granulosa cells and secreted throughout the menstrual cycle [2]. A strong correlation exists between AFC and AMH [4], and thus could possibly be used interchangeably in estimating ovarian reserves [5].

Data have demonstrated that AFC and AMH decrease with increasing age, reflecting a decline of the non-growing follicle pool [2]. However, it is still not clear about the rate at which it declines with age and varies across populations. Some studies have reported a linear age-related decline in AFC [1, 6–8]; while others have proposed that the fall is biphasic [9–11]. Similarly, linear [11] and non-linear [12–14] age-related decline patterns had also been observed for AMH. A previous report evaluating ovarian reserve has shown that variations in AFC and AMH were observed between different age-matched ethnic populations, suggesting race-dependent differences in ovarian aging [15].

A local sonographic study of AFC and AMH is important to provide valuable information on the status of ovarian reserve for women who are seeking infertility treatment. Centiles for AFC-age and AMH-age nomograms are useful for pretreatment counseling purpose by informing the women about their reproductive performance [10, 16] and serve as the basic principle to determine the appropriate procedure for women in the field of assisted reproductive technology (ART). By considering ethnicity-specific effect on the age-related declines in AFC and AMH which has been suggested as an important factor in ART [15], we sought i) to determine the relationships of AFC and AMH with age; ii) to examine the relation between AFC and AMH; and iii) to construct age-related nomograms for AFC and AMH among subfertile Chinese women.

## Materials and methods

This is a retrospective observational study involving Chinese women who had their total AFC and serum AMH measured as part of their subfertility screening from December 2010 until November 2014 in KK Women’s and Children’s Hospital, Singapore. Women typically were unable to conceive after twelve months of trying-to-conceive without contraception, and were referred from their primary care doctors for further management. An ethical approval was obtained from the Centralised Institutional Review Board of SingHealth (2014/705/D). All data were analyzed anonymously; therefore, informed consent for each individual was neither necessary nor possible.

To assess AFC, women underwent transvaginal sonography based on three-dimensional acquisition and semi-automated technique using the AFC function from the Voluson E8 ultrasound machine (GE Healthcare, United States) in the early follicular phase of the menstrual cycle. The number of antral follicles of 2 to 8mm in diameter was counted. The follicle counts and sizes generated by the ultrasound machine were manually checked by the radiologist and corrected if required. Total AFC values were categorized as low (<7), normal (7–16) and high (>16) [5]. Serum AMH levels were measured using the AMH Gen II ELISA (Beckman Coulter, US). Performance of the assay kit was validated by in-house assessment of reproducibility and linearity. Within-laboratory, inter-day coefficient of variation (CV) was 5.0% at 2.8 ng/ml and 5.9% at 8.9 ng/ml. Linearity of the assay between 0.1 and 21.0 ng/ml was confirmed using a 5-point dilution protocol, whereby means of duplicate measurements were accepted when they were within 10% of the target concentrations. Serum samples with AMH levels above 21.0 ng/ml were diluted 1:4 using diluent buffer before analysis according to the manufacturer’s protocol. We categorized serum AMH levels into low (<1.36 ng/ml), normal (1.36–4.00 ng/ml) and high levels (>4.00 ng/ml) [5]. Records on total AFC and AMH levels were extracted from the central database system, KK Women’s and Children’s Hospital.

### Statistical analysis

The relationships of AFC and AMH with age were examined using ordinary least squares regression. The relations between raw and categorized AFC and AMH were assessed using Spearman’s correlation coefficient (*r*) and weighted Kappa test (*k*), respectively. The statistical analyses were performed using IBM SPSS statistics, Version 20 (USA) and Stata 13.1 (USA).

The age-specific AFC centile chart was generated using LMS Chartmaker Light software version 2.54 (Medical Research Council, UK). It produced a model that expressed the centiles in terms of age-specific curves called L (skewness), M (median) and S (coefficient of variation). The equivalent degrees of freedom (EDFs) for the L, M and S parameters were adjusted to obtain a model with minimal deviance. Seven empirical centiles, including the 3rd, 10th, 25th, 50th, 75th, 90th and 97^th^ centiles, and nomogram tables were constructed.

## Results

A total of 1,015 Chinese women underwent subfertility screening with total AFC and serum AMH levels being measured. Among them, six were excluded from our analysis due to extreme age with small sample size (age <26 years, n = 2; age >44 years, n = 4) that could reduce accuracy and reliability while producing nomogram. The remaining 1,009 Chinese women were included in this study, with age range of 26 to 44 years (mean 35.4 years; SD 3.7). The means (SDs) of AFC and AMH were 16.72 (9.25) and 4.18 ng/mL (3.72), respectively.

**Fig 1:**
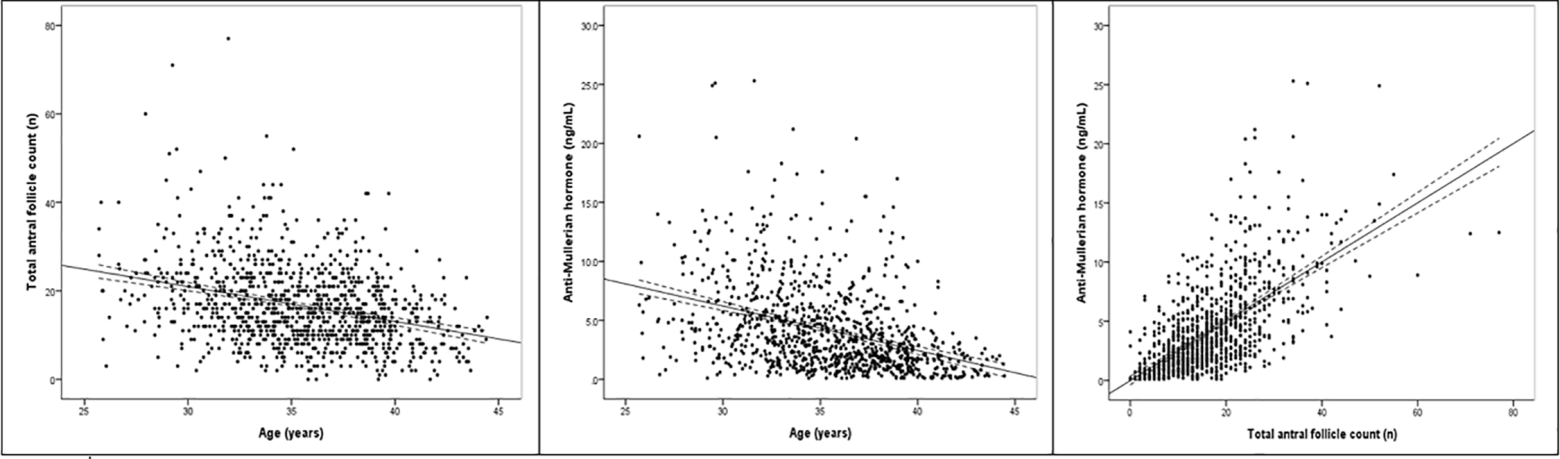
Relations between total antral follicle count (AFC), Anti-Mullerian hormone (AMH) and age (n = 1009). The dotted lines indicate 95% confidence interval. Left, relation between total AFC and age (y = -0.79x + 44.60; R^2^ = 10.1%). Middle, relation between AMH and age (y = -0.38x + 17.50; R^2^ = 14.3%). Right, relation between total AFC and AMH (y = 1.55x + 10.23; R^2^ = 38.8%).

Relations between AFC, AMH and age are shown in Fig 1. On average, AFC and AMH decreased respectively by 0.79 follicle (95% confidence interval -0.93, -0.64) and 0.38 ng/mL (95% confidence interval -0.43, -0.32) per year of age. Age alone accounted for 10.1% of the variation (R^2^) in AFC and 14.3% of the variation in AMH. When AFC and AMH were categorized into different levels [5], it was found that 10.1% women had low AFC, followed by normal (45.7%) and high values (44.2%); while 21.2% women had low AMH, followed by 39.6% and 39.1% women with normal and high AMH levels. Based on weighted Cohen’s Kappa, a fair agreement was found between AFC and AMH categories (*k* = 0.39); while modest correlation was observed between AFC and AMH (*r* = 0.62, p<0.001).

**Fig 2:**
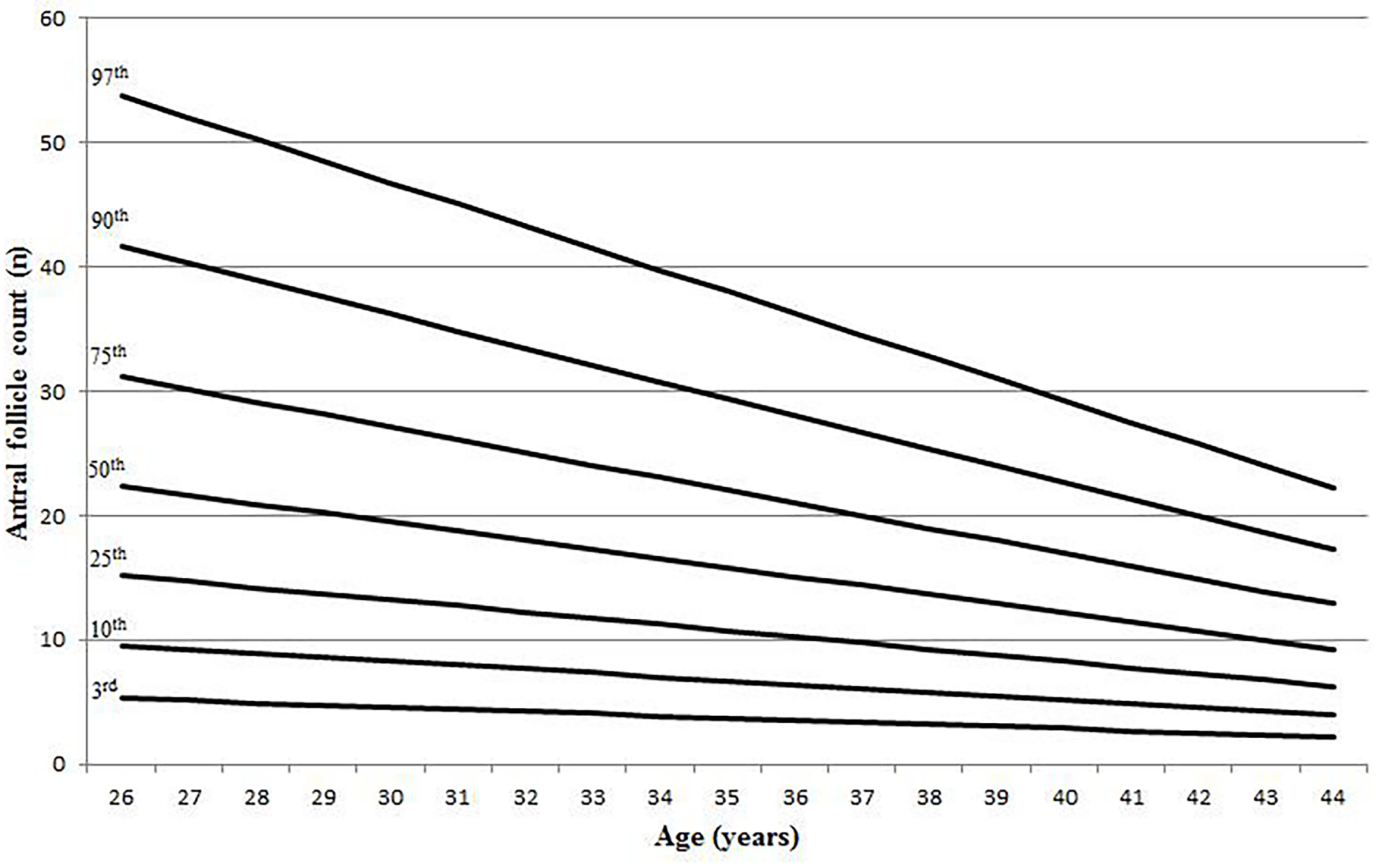
Centiles of antral follicle count by age.

**Fig 3:**
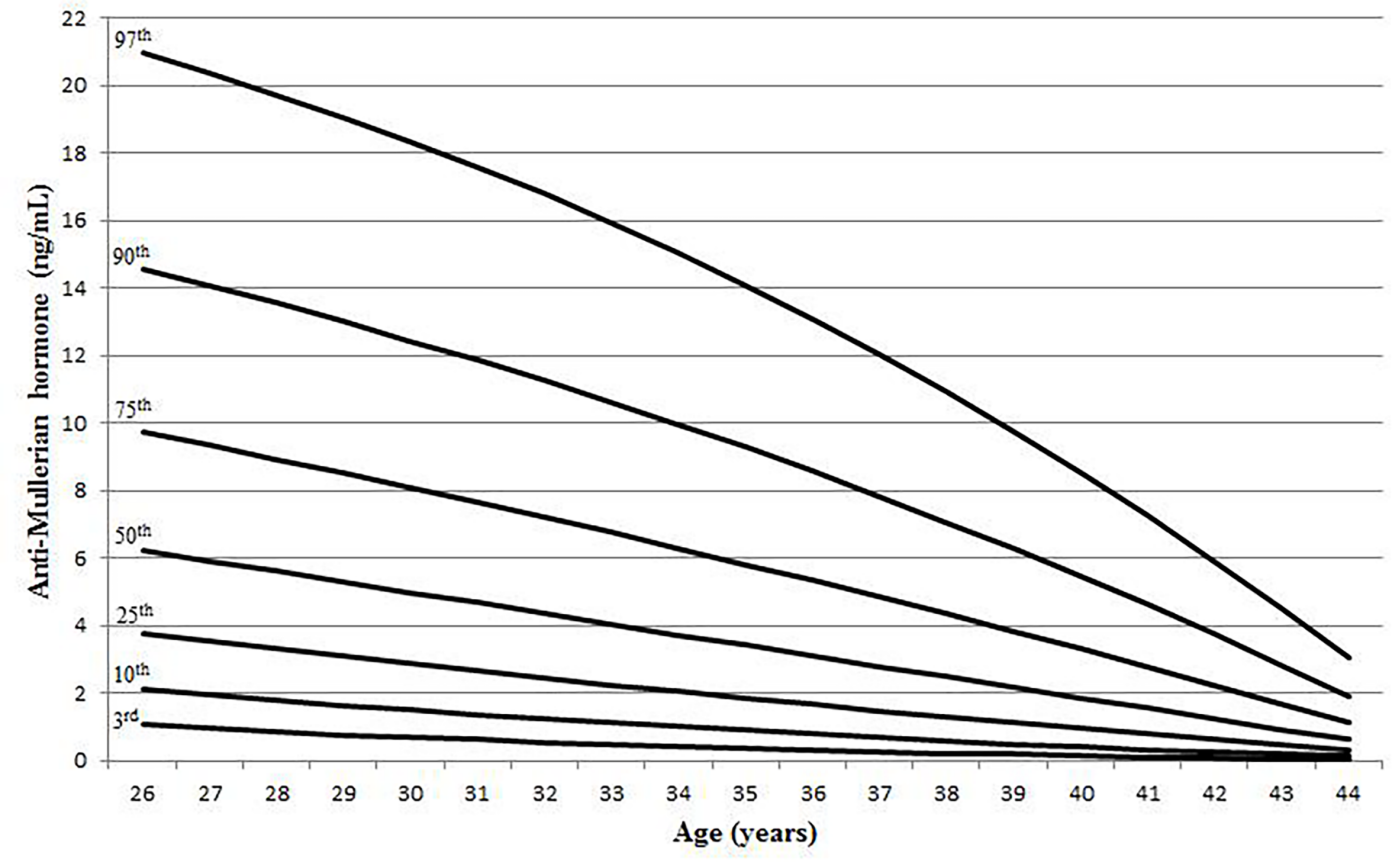
Centiles of anti-Mullerian hormone by age.

The lambda-mu-sigma (LMS) model demonstrated a linear decline in AFC with age. It was found that the decreasing pattern is quite similar for all percentiles (Fig 2). AFC values of 3^rd^, 10^th^, 25^th^, 50^th^, 75^th^, 90^th^ and 97^th^ centiles as a function of age are shown in Table 1. For AMH, the pattern is linear for the 75^th^ percentile and below but biphasic for the 90^th^ and 97^th^ percentile. The rate of AMH decline was slower from 26 to 30 year-old and accelerated afterwards (Fig 3). AMH levels of 3^rd^, 10^th^, 25^th^, 50^th^, 75^th^, 90^th^ and 97^th^ centiles as a function of age are shown in Table 2. Overall, there were large inter-individual variations in AFC and AMH up to 40 year-old.

**Table 1:** Antral follicle count by centile and age.

Age	3^rd^	10^th^	25^th^	50^th^	75^th^	90^th^	97^th^
26	5.30	9.54	15.21	22.41	31.19	41.63	53.79
27	5.12	9.23	14.72	21.68	30.18	40.28	52.04
28	4.95	8.92	14.22	20.95	29.16	38.92	50.29
29	4.78	8.61	13.73	20.22	28.15	37.57	48.53
30	4.61	8.30	13.23	19.49	27.13	36.21	46.78
31	4.43	7.99	12.74	18.76	26.12	34.86	45.03
32	4.26	7.67	12.24	18.03	25.10	33.50	43.28
33	4.09	7.36	11.75	17.30	24.08	32.15	41.53
34	3.92	7.05	11.25	16.57	23.07	30.79	39.78
35	3.74	6.74	10.76	15.84	22.05	29.43	38.03
36	3.57	6.43	10.26	15.11	21.04	28.08	36.27
37	3.40	6.12	9.77	14.38	20.02	26.72	34.52
38	3.23	5.81	9.27	13.65	19.01	25.37	32.77
39	3.05	5.50	8.77	12.92	17.99	24.01	31.02
40	2.88	5.19	8.28	12.19	16.97	22.66	29.27
41	2.71	4.88	7.78	11.46	15.96	21.30	27.52
42	2.54	4.57	7.29	10.73	14.94	19.94	25.77
43	2.36	4.26	6.79	10.01	13.93	18.59	24.02
44	2.19	3.95	6.30	9.28	12.91	17.23	22.26

**Table 2:** Anti-Mullerian hormone by centile and age.

Age	3^rd^	10^th^	25^th^	50^th^	75^th^	90^th^	97^th^
26	1.06	2.10	3.76	6.23	9.75	14.56	20.96
27	0.96	1.94	3.53	5.92	9.35	14.07	20.37
28	0.87	1.79	3.30	5.61	8.94	13.55	19.74
29	0.78	1.64	3.08	5.30	8.52	13.01	19.07
30	0.69	1.50	2.87	4.98	8.09	12.45	18.35
31	0.62	1.37	2.65	4.67	7.65	11.86	17.60
32	0.55	1.24	2.45	4.36	7.21	11.26	16.79
33	0.48	1.12	2.24	4.05	6.75	10.62	15.94
34	0.42	1.00	2.05	3.74	6.29	9.96	15.04
35	0.36	0.89	1.85	3.42	5.82	9.28	14.08
36	0.31	0.79	1.66	3.11	5.34	8.57	13.08
37	0.27	0.69	1.48	2.80	4.84	7.84	12.03
38	0.22	0.59	1.30	2.49	4.34	7.08	10.92
39	0.18	0.50	1.12	2.17	3.83	6.29	9.76
40	0.15	0.42	0.95	1.86	3.31	5.47	8.54
41	0.12	0.34	0.78	1.55	2.78	4.63	7.26
42	0.09	0.26	0.61	1.24	2.24	3.76	5.92
43	0.06	0.19	0.45	0.93	1.69	2.85	4.52
44	0.04	0.12	0.30	0.61	1.13	1.92	3.06

## Discussion

This study examined the relationship of total AFC and AMH with age among Chinese women suffering from subfertility. The customized nomograms created aim at facilitating age-specific interpretation of the two commonly used ovarian reserve markers and therefore establish tailored therapeutic recommendations for women undergoing ART. It was found that the AFC decreases by 0.79 follicles per year of age and AMH decreases by 0.38 ng/mL per year of age. The results revealed that AFC and AMH generally decline in a linear fashion with increasing age, although there is a slight non-linear pattern observed for AMH in the 90^th^ and 97^th^ percentiles. These data showed that the ovarian reserve for older patients decreases at a constant rate over time. Faster decline rates of AFC and AMH were observed in higher centiles.

The rate of decline in measured AFC varies according to studied populations, with a reported estimated loss of 0.35 to 0.97 follicle per year [1, 6–8, 17]. Our study revealed that the decline of AFC in subfertile Chinese women was 0.79 follicles per year, which is higher than the estimated loss of 0.47 follicles per year reported in subfertile Taiwanese Chinese women, with a similar linear decline pattern of AFC with age to ours [7]. In contrast, few studies found a linear biphasic relation between AFC and age, with ‘switching age’ being identified at certain time point where the follicles loss is accelerated [10, 11]. The reasons or mechanisms to explain the differences between populations in AFC decline are unclear. Many genetic and environmental factors that influence follicle loss had not yet been identified [11].

The declining pattern of AMH with age as shown in our study is similar with the findings from Wiweko et al. (2013) among 1,616 infertile Indonesian women [11]. The study demonstrated that a generally linear decreasing pattern was found for AMH with age in the 75^th^ percentile and below, whereas a slight biphasic pattern was observed in the 90^th^ and 97^th^ percentiles which could be due to the inclusion of women with polycystic ovarian syndrome [11]. However, studies by Nelson et al. (2011) [12] and Seifer et al. (2011) [18] among infertile Caucasian women showed that AMH declined in a non-linear pattern with age, which was best described by a quadratic equation; while the most recent study by Naasan et al. (2015) [19] found that AMH declined in a linear biphasic fashion with age. As compared to AFC, AMH had a stronger degree of relationship with age based on the R^2^ values. Nevertheless, there is still a considerable amount of variation with AFC and AMH that cannot be explained by age alone.

Historically, the decline in female fertility was predicted to occur in a biphasic-exponential fashion [9], but recently it is evidenced that the decreasing fertility could follow a gradual decline or linear pattern [17, 20, 21]. This is supported by Hansen et al. (2008) [22] who challenged the theoretical relationship between non-growing follicles and age in a histopathology study, reporting there is no abrupt change in oocyte number but rather a subtle change in decay over age. Rosen et al. (2012) indicated that the only markers that followed the pattern of oocyte loss observed histologically were AFC and AMH [14]. Our results provide additional supportive evidence showing a linear declining pattern of AFC and AMH with advancing age, which was confirmed by the LMS model. This trajectory of AFC and AMH with age could be best described as a constant loss of antral follicles throughout reproductive period. Such finding has a pronounce influence on clinical decision making in fertility management of older women. It suggests that practitioners should not change fertility management with the perception that there is a dramatic decline in AFC and AMH at a certain age.

Recent systematic review reported that AFC and AMH predict the whole spectrum of ovarian response with reliable accuracy, suggesting the use of either marker interchangeability in clinical practice [5]. In the present study, we observed a modest correlation and a fair agreement between AFC and AMH levels, indicating considerable differences between these two markers with regard to ovarian reserve which could be due to distinct influential factors. AFC was shown to be generally reduced with cigarette smoking in women [23], while AMH was found to be lower in overweight/ obese women [24]. These findings suggest that both AFC and AMH should be evaluated simultaneously, meanwhile consider related influential factors when estimating ovarian reserve and potential ovarian responses.

We acknowledge a few limitations in our study. The present study is mainly restricted by its cross-sectional design. A longitudinal prospective study will be required to best address the relationship of AFC and AMH with increasing chronological age, and validate our nomograms. This information is useful to improve our understanding of reproductive aging due to its important implications for pregnancy planning and infertility treatment decisions. Another limitation is that the dataset is only restricted to Chinese population though Singapore is composed of multiethnic groups (e.g. Malays and Indians). As there could be a potential ethnic variation in the markers of ovarian reserve [15], we decided not to include dataset from other ethnic groups with limited sample size available (70% of our population being Chinese). Observations that reported in this study was based on a relatively small sample size (n = 1015), where the results might not provide a high level of evidence as in other large population-based studies. Additional factors that can influence AFC and AMH levels, such as body mass index, genetic background, the use of exogenous hormone and medications, presence of other diseases and environmental contributors [2] were uncharacterized and therefore, could not be assessed. Finally, the present findings should be interpreted cautiously due to the existence of heterogeneity among subfertile populations with regards to aetiology. Despite these limitations, the age-specific AFC and AMH nomograms as shown in this study provide helpful information for local practitioner and patients in either a fertility clinic setting, or that in the prediction of menopause among Chinese women.

In summary, we have shown that the declines of AFC and AMH over age were generally linear among subfertile Chinese women in Singapore. The age-related AFC nomogram produced in this study could serve as a reference guide in subfertile women to tailor their ovarian stimulation regime. However, future validation of the nomograms with longitudinal data is required.
